# On the Relation Between Over-Indebtedness and Well-Being: An Analysis of the Mechanisms Influencing Health, Sleep, Life Satisfaction, and Emotional Well-Being

**DOI:** 10.3389/fpsyg.2021.591875

**Published:** 2021-04-29

**Authors:** Mário B. Ferreira, Filipa de Almeida, Jerônimo C. Soro, Márcia Maurer Herter, Diego Costa Pinto, Carla Sofia Silva

**Affiliations:** ^1^CICPSI, Faculdade de Psicologia, Universidade de Lisboa, Lisbon, Portugal; ^2^Faculdade de Psicologia, Universidade de Lisboa, Lisbon, Portugal; ^3^Católica Lisbon School of Business and Economics, Universidade Católica Portuguesa, Lisbon, Portugal; ^4^Business and Law Research Unit, Universidade Europeia, Lisbon, Portugal; ^5^NOVA Information Management School (NOVA IMS), Universidade Nova de Lisboa, Lisbon, Portugal

**Keywords:** debt, over-indebtedness, subjective well-being, life satisfaction, net affect

## Abstract

This paper aims to explore the association between over-indebtedness and two facets of well-being – life satisfaction and emotional well-being. Although prior research has associated over-indebtedness with lower life satisfaction, this study contributes to the extant literature by revealing its effects on emotional well-being, which is a crucial component of well-being that has received less attention. Besides subjective well-being (SWB), reported health, and sleep quality were also assessed. The findings suggest that over-indebted (compared to non-over-indebted) consumers have lower life satisfaction and emotional well-being, as well as poorer (reported) health and sleep quality. Furthermore, over-indebtedness impacts life satisfaction and emotional well-being through different mechanisms. Consumers decreased perceived control accounts for the impact of over-indebtedness on both facets of well-being (as well as on reported health and sleep). Financial well-being (a specific component of life satisfaction), partly mediates the impact of indebtedness status on overall life satisfaction. The current study contributes to research focusing on the relationship between indebtedness, well-being, health, and sleep quality, and provides relevant theoretical and practical implications.

## Introduction

Being indebted at certain stages of life may be a means of consumption smoothing across the lifecycle, improving households’ economic welfare (e.g., Hall, 1978; Lombardi et al., 2017). However, debt becomes most problematic when it leads to over-indebtedness, that is, when it exceeds household resources leading to the inability to meet all payment obligations and to cover living expenses over long time periods. Indeed, the burden of over-indebtedness has been shown to have a negative impact on several indicators of psychological and physical health.

This study seeks to contribute to the extant literature by focusing on the relationship between debt and subjective well-being (SWB). Specifically, we explore how being over-indebted (vs. not being over-indebted) affects two different facets of SWB: life satisfaction and emotional well-being. In addition, the current study also compares over-indebted and non-over-indebted consumers on measures of reported health and sleep quality.

Finally, the mediating role of (a) consumers’ financial well-being (a specific component of life satisfaction); (b) financial anxiety; and (c) perceived sense of control, is explored in order to look for empirical evidence on the underlying processes of the relationship between debt and SWB as well as the relationship between debt and measures of health and sleep quality.

In what follows, we begin by briefly reviewing different concepts of over-indebtedness. We then notice that while there is a substantial amount of literature on the detrimental effects of over-indebtedness, there is a relative shortage of studies on the impact of over-indebtedness on SWB. Finally, the goals and hypotheses of the study here reported are presented.

Several different definitions of over-indebtedness and consequently different ways on how to measure it have been proposed in the literature (Berthoud and Kempson, 1992; Bridges and Disney, 2004; Kempson et al., 2004; European commission, 2008; D’Alessio and Iezzi, 2013; Angel, 2016). Nevertheless, research has been converging on a shared set of indicators (Keese, 2009; BIS–Department for Business, Innovation and Skill, 2010) that broadly refer to four features of over-indebtedness: making high repayments relative to income (e.g., households spending more than 30% of their gross monthly income on unsecured repayments), having a high number of credit commitments (e.g., four or more credit loans), being in arrears, and the subjective perception of debt as a burden (D’Alessio and Iezzi, 2013).

All of these indicators of debt difficulties provide potentially valuable information but suffer from some drawbacks. The first two set fixed limits – of repayment-to-income ratios and of number of credit commitments – to define and measure over-indebtedness. However, such limits depend on income level and household assets. For instance, debt (relative to income) and number of credits can increase above defined limits for households with high levels of income, without this necessarily making debt management problems more acute.

By using information on household arrears in making payments, the third feature is less vulnerable to the problem of setting arbitrary limits. However, the definition of the point where over-indebtedness begins based on arrears depends on the judged seriousness of the arrears, which, in turn, is likely to be dependent on the financial circumstances of the household, among other variables.

Given these limitations associated with the first three indicators, D’Alessio and Iezzi (2013) argued that a better way to identify over-indebtedness may be to enquire households on whether or not they are facing debt repayment difficulties. The downside of this type of subjective indicator is that the interpretation of terms such as “heavy repayment difficulties” is likely to vary among households (Drentea and Lavrakas, 2000) and to be sensitive to consumer’s individual differences.

Finally, other objective indicators of over-indebtedness, such as judicial decisions declaring personal bankruptcy (or other court arranged solutions for resolution), are likely to be too narrow in the identification of over-indebted households failing to capture several, if not most, circumstances of over-indebtedness (D’Alessio and Iezzi, 2013).

One way to attenuate this problem is to check for the existence of more than one indicator when looking into potential cases of over-indebtedness (Białowolski, 2019). In the study, here presented, over-indebted households are consumers who voluntary looked for the help of debt advise experts, reporting an inability to meet recurrent expenses, and who in general spent more than 30% of their gross monthly income on total borrowing repayments (secured and unsecured).

The reviewed indicators of over-indebtedness mostly refer to the process of becoming over-indebted, rather than the outcomes associated with having problems with debts. However, being over-indebted has considerable negative impacts on households. From an economical perspective, over-indebted households often face liquidity constrains (Attanasio, 1995; Crook, 2003) as they become unable to borrow against future earnings, making it increasingly challenging to accommodate their financial needs.

From a socio-psychological perspective, individuals with unmet loan payments have been shown to display more suicidal ideation and are at a higher risk of depression than those without such financial difficulties (e.g., Hintikka et al., 1998; Gathergood, 2012; Turunen and Hiilamo, 2014). Unpaid financial obligations have also been associated with poorer subjective health, deterioration of health-related behavior, and physical illness (Lenton and Mosley, 2008; Bridges and Disney, 2010; Chmelar, 2013; Guiso and Sodini, 2013; Sweet et al., 2013; Turunen and Hiilamo, 2014; Clayton et al., 2015). Confirming this pattern, a recent longitudinal study of Finnish adults found an association between over-indebtedness and an increased incidence of various chronic diseases (Blomgren et al., 2016). More recently, Warth et al. (2019) found a negative relationship between over-indebtedness and sleep quality. Notably, poor sleep plays a major role in a variety of health problems, from hypertension (Gangwisch et al., 2006; Buxton and Marcelli, 2010; Meng et al., 2013) to diabetes (Buxton and Marcelli, 2010; Morselli et al., 2012; Zizi et al., 2012; Grandner et al., 2014) and mortality (Gallicchio and Kalesan, 2009; Grandner and Patel, 2009; Cappuccio et al., 2010; Grandner et al., 2010).

Taken together, such detrimental consequences are worrisome given the increasing number of over-indebted households across Europe and around the world (e.g., Betti et al., 2007; Barba and Pivetti, 2009; Harvey, 2011; Kempson, 2015), and serve to highlight the importance of further research to better understand the relationship between over-indebtedness and different indicators of well-being and health.

While there is a large body of literature on the risk factors, remedies, and detrimental effects of over-indebtedness, there is a relative lack of research on the impact of debt on SWB. This is surprising given the longstanding research interest in the relationship between finances and SWB (e.g., Diener and Biswas-Diener, 2002; Howell and Howell, 2008). Recently, Tay et al. (2017) conducted a systematic review and meta-analysis of the literature on debt and different aspects of SWB, including overall well-being (e.g., life satisfaction), domain-specific (e.g., financial well-being), and emotional well-being (i.e., positive and negative feelings). Although only a relatively small number of empirical studies were found, results suggest a negative, but somewhat weak association between debt and SWB (but see Białowolski et al., 2019). Several reasons may contribute to this. First, only seven studies of the 19 identified by Tay et al. (2017) met their criteria for meta-analysis. Second, according to the hedonic treadmill hypothesis (Brickman and Campbell, 1971), people rapidly adapt to change. This suggests that the deteriorated life circumstances associated with indebtedness could have an attenuated effect on life satisfaction with the passage of time. Indeed, prior research indicates that most life circumstances quickly cease to influence global reports of SWB (Easterlin, 1995; Kahneman et al., 2004).

Third, Tay et al. (2017) did not distinguish between being the holder of manageable debt and being over-indebted (although their meta-analysis showed that variables, such as level of debt and overall financial resources, play a critical role as moderators of the relationship between debt and SWB).

In line with other research (Angel, 2016; Białowolski et al., 2019), the present study considers this crucial distinction, as being over-indebted is not merely a function of debt but it may involve, as aforementioned, several other features. Moreover, since over-indebtedness is often associated with careless consumer behavior and financial imprudence (Disney et al., 2008; Anderloni and Vandone, 2010), being over-indebted is often a source of social stigma and prejudice, which puts additional pressure on these consumers’ already difficult living conditions.

Furthermore, although prior research (e.g., O’Neill et al., 2005; Zhang and Kemp, 2009; Drentea and Reynolds, 2012; Hogan et al., 2013; Olson-Garriott et al., 2015; see Tay et al., 2017, for a review) has used different measures of well-being to understand how people think and feel about their lives, it has not clearly distinguished between the impact of over-indebtedness on two qualitatively different facets of SWB: life satisfaction (based on a global evaluation by the individual of his/her life) and emotional well-being (the affect experienced by an individual on a more day-to-day basis; Pavot and Diener, 1993; Kahneman and Riis, 2005). Nonetheless, life satisfaction and emotional well-being are different constructs with moderate to high discriminant validity (Lucas et al., 1996; see also Dolan et al., 2017). For instance, United States consumers who earn above $75,000 annually are increasingly more satisfied with their lives (life satisfaction) but they do not have higher emotional well-being (based on experienced feelings; Kahneman and Deaton, 2010).

In this paper, the effect of being over-indebted (vs. not being over-indebted) on life satisfaction and emotional well-being is explored. In addition, the current study also includes measures of global subjective health and sleep quality in which over-indebted and non-over-indebted consumers are also compared. The goal is to ascertain whether over-indebted consumers show lower levels of SWB (both life satisfaction and emotional well-being) than non-over-indebted consumers. Based on prior cited research, over-indebted consumers are further expected to have poorer reported health and sleep quality, in addition to increased sleep-related disturbances, than their non-over-indebted counterparts.

Furthermore, two possible but not mutually exclusive accounts are considered for why over-indebted consumers might show lower SWB (as well as lower quality of health and sleep) than non-over-indebted consumers. First, given that financial well-being is one of the key life domains that inform SWB (Diener et al., 1999; Kahneman, 1999), becoming over-indebted may adversely affect financial well-being, and thus contribute to decreasing SWB. However, since financial well-being is defined as personal satisfaction with one’s financial status (i.e., a specific component of life satisfaction), the mediating role of financial well-being is expected to occur for life satisfaction but not for emotional well-being.

Second, being indebted greatly limits the extent to which consumers may attain their life goals, calling into question the fulfillment of fundamental needs of autonomy and self-control, which are crucial for promoting SWB (Sheldon et al., 2010; Tay and Diener, 2011). Hence, by leading to the depletion of financial resources, over-indebtedness may not only create financial anxiety but also reduce consumers’ perceived self-control over their own lives. Both aspects (financial anxiety and reduced self-control) could be expected to lower SWB.

Since financial anxiety and perceived self-control are likely to affect not only the global attainment of one’s life goals but also consumers’ daily emotional experience, these factors are expected to mediate both life satisfaction and SWB.

We further expect to find that financial satisfaction, perceived control, and financial anxiety mediate the relationship between over-indebtedness and both health and sleep. Such expectation is in line with previous literature. Indeed, financial satisfaction has been found to be associated to both health (Kostelecky, 1994; Hsieh, 2001; Hansen et al., 2008) and sleep quality (Summers and Gutierrez, 2018). Perceived control has also been shown to have a significant impact on health and sleep (Bobak et al., 1998; Bosma et al., 1999; Gerstorf et al., 2011; Adachi et al., 2013; Gould et al., 2016). Finally, stress (Keller et al., 2012) and anxiety (Gould et al., 2016) both have a negative impact on one’s health and sleep.

## Materials and Methods

### Participants

Three hundred and sixty-five Portuguese consumers responded to the study questionnaire, of which 236 were over-indebted and 129 were non-over-indebted. The questionnaire was created and applied in the context of a research project on over-indebtedness funded by the Portuguese science foundation with the collaboration of the debt advice department of an NGO for the Consumer Defense (DECO). DECO’s debt advice experts offer counseling to over-indebted households who contact them free of charge. Households may contact DECO online (by email or through their website) or directly in their offices. Over-indebted consumers (i.e., consumers reporting an inability to meet recurrent expenses, and who in general spent more than 30% of their gross monthly income on total borrowing repayments – secured and unsecured) who contacted DECO between 2017 and 2018 were invited to voluntarily participate in the study by filling the questionnaire. Data from non-over-indebted consumers were collected during the same time period through convenience sampling from different sources (e.g., part-time courses on different areas of education and development promoted by the city governments and other NGO platforms of social action). Six participants of this sample reported being over-indebted and their data were included in the sample of over-indebted consumers. A summary of the socio-demographic measures of both over-indebted and non-over-indebted groups are displayed in Table 1. Apart from variables pertaining to debt, which were expected to differ between the groups, income, and education also differed significantly between the over-indebted and the non-over-indebted groups.

**Table 1:** Socio-demographic characteristics of over-indebted and non-over-indebted samples of participants.

	Over-indebted	Non-over-indebted
**Age**		
*M* (*SD*)	52.30 (11.66)	48.93 (17.61)
Valid N	86	128
Income (monthly) in Euros^**^		
*M* (*SD*)	1100.65 (562.54)	2103.50 (2176.07)
Valid N	154	120
Income (monthly) per capita in Euros^**^		
*M* (*SD*)	597.87 (372.34)	1042.84 (825.00)
Valid N	147	119
Debt in Euros^**^		
M (SD)	733.88 (944.51)	170.26 (222.74)
Valid N	149	113
Debt to income ratio^*^		
*M* (*SD*)	0.83 (1.89)	0.20 (0.17)
Valid N	140	55
Debt+expenses to income ratio^*^		
*M* (*SD*)	1.61 (2.19)	0.81 (0.50)
Valid N	83	55
People in the household^*^		
*M* (*SD*)	2.10 (1.00)	2.40 (1.27)
Valid N	152	126
**Schooling (absolute and relative frequencies)**		
1st cycle (6–9 years old)	20 (12.82%)	3 (2.34%)
2nd cycle (10–11 years old)	13 (8.33%)	8 (6.25%)
3rd cycle (12–14 years old)	35 (22.43%)	26 (20.31%)
Secondary and Vocational ed. (15–17 years old)	63 (40.38%)	42 (32.81%)
Higher education	25 (16.03%)	49 (38.28%)
Valid N	156	128
**Marital status (absolute and relative frequencies)**		
Single	37 (23.56%)	31 (24.21%)
Divorced/Separated	45 (28.66%)	23 (17.97%)
Married/Domestic partnership	64 (40.76%)	54 (42.18%)
Widowed	11 (7%)	20 (15.62%)
Valid N	157	128
**Professional status (absolute and relative frequencies)**		
Unemployed	31 (19.87%)	18 (14.4%)
Informal jobs	3 (1.92%)	7 (5.6%)
Retired	34 (21.79%)	41 (32.8%)
(Self-)Employed	88 (56.41%)	59 (47.20%)
Valid N	156	125

The debt is the total paid monthly for all types of credit.

* *p* < 0.05;

** *p* < 0.001.

### Materials

The questionnaire included (a) socio-demographic items (e.g., marital status, schooling, professional status, and number of people in the household); (b) questions on financial aspects of the respondent’s life (e.g., income, monthly expenses, and monthly installments of loans); and (c) the main dependent measures of the current study: life satisfaction, emotional well-being, sleep quality, and reported health. Perceived control, financial anxiety, and financial well-being were also assessed as potential mediators. Finally, the questionnaire included other measures collected for different research purposes, which will not be addressed herein.

#### Life Satisfaction

Life Satisfaction was assessed using the Cantril Self-Anchoring Striving Scale (Cantril, 1965). Participants were asked to imagine a ladder with steps from 0 to 10, where 0 was the worst possible life for them and 10 the best possible life for them. They were then asked to indicate the step on which they saw their lives at that particular time and the step on which they envisaged their lives 5 years from then.

#### Emotional Well-Being

Emotional well-being was assessed using a simplified version of the Day Reconstruction Method (DRM – Kahneman et al., 2004), in which participants were required to indicate on a five-point rating scale (1 – not at all, 5 – extremely) how intensely they had felt a set of negative and positive emotions (used in the original DRM questionnaire) during the morning, afternoon, and evening of the previous day. The negative emotions included: frustrated, angry, depressed, hassled, and criticized. The positive emotions included: happy and competent. Mean responses for positive and negative emotions were obtained for each moment of the day and in total. Four scores of net affect (composed of the subtraction of the mean negative emotions from the mean positive ones) were computed, one for each part of the day (morning, afternoon, and evening) and one global score (entire day). Positive values represent positive net affect, and negative values represent negative net affect (Kahneman et al., 2004).

#### Reported Health

Health was evaluated by means of two questions: “Globally, how do you evaluate your health?” and “How do you evaluate your health compared to other people of your age?” based on the same rating scale (1 – Excellent, 2 – Good, 3 – Fair, 4 – Poor, and 5 – Very poor). Individual responses to these questions were averaged in a composite measure of self-reported health (Cronbach’s alpha = 0.91).

#### Sleep Quality

Subjective quality of sleep and sleep-related disturbances were measured using four questions from the Pittsburgh Sleep Quality Index (PSQI – Buysse et al., 1989). Three components of the PSQI were assessed: subjective sleep quality (“During the past month, how would you rate your sleep quality overall?,” using a four-point rating scale: 1 – Very good, 2 – Fairly good, 3 – Fairly bad, and 4 – Very bad); sleep duration (“During the past month how many hours of actual sleep have you managed to get at night? – This may differ to the number of hours you have spent in bed,” by means of the following four-point rating scale: (>7 h, 6–7 h, 5–6 h, and <5 h – where >7 = 1, 6–7 = 2, 5–6 = 3, and <5 = 4); and daytime dysfunction (“During the past month, how often have you had trouble staying awake while driving, eating meals, or engaging in social activity?,” measured with the following rating scale: 1 – Not at all during the past month; 2 – Less than once a week; 3 – Once or twice a week; and 4 – Three or more times a week and “During the past month, how much of a problem has it been for you to keep up enough enthusiasm to get things done?,” measured with the following scale: 1 – No problem at all; 2 – Only a slight problem; 3 – Somewhat of a problem; and 4 – A very big problem). A confirmatory factor analysis was performed to evaluate how well these four items defined an underlying sleep quality factor. Results of this analysis revealed a good model fit, *χ*^2^(2) = 9.18, *p* = 0.01; CFI = 0.98; TLI = 0.94; SRMR = 0.03). All factor loadings were highly significant (i.e., *p* < 0.001) and higher than 0.50. We then used the standardized factor loadings to calculate the composite reliability or coefficient omega (Ω), which was above the 0.70 benchmark for acceptable reliability (Ω = 0.78; Nunnally and Bernstein, 1994).

#### Perceived Control

Perceived control was measured with two questions adapted from the perceived stress scale of Cohen et al. (1983): “How often have you been feeling you do not have control over the important things in your life?” and “How often have you been feeling you are not able to deal with everything you have to do?” (Both using the following rating scale: 1 – Never, 2 – Hardly ever, 3 – Sometimes, 4 – Frequently, and 5 – Very frequently). Individual responses to these questions were averaged in a composite measure of perceived control (Cronbach’s alpha = 0.82).

#### Financial Anxiety

Financial anxiety was assessed using nine items from the Financial Anxiety Scale (FAS; Shapiro and Burchell, 2012, Study 1). Participants responded to the statements on a five-point rating scale from 1 (Totally disagree) to 5 (Totally agree). Examples of the FAS are: “Thinking about my personal finances can make me feel guilty,” “Thinking about my personal finances can make me feel anxious,” and “Discussing my finances can make my heart race or make me feel stressed” (Cronbach’s alpha = 0.86).

#### Financial Well-Being

Financial well-being was assessed by questioning participants on how satisfied they were with their financial status compared to their friends. Participants answered using a rating scale from 0 (Not satisfied at all) to 10 (Very satisfied).

### Procedure

Over-indebted consumers responded to the questionnaire either in a paper form (while waiting for their individual appointment with DECO experts) or in an editable computer file sent to them by email (those who contacted the consumer defense association through its website or by email). Non-over-indebted consumers responded to the questionnaire on paper. Participants did not always respond to all the questions. As a consequence, there is some variation in the number of participants for each analysis. The questionnaire was approved by the ethics committee of the Faculdade de Psicologia of Universidade de Lisboa. Data from consumers were used with their informed consent and always anonymously.

## Results

### Comparing Between Over-Indebted and Non-over-Indebted Consumers

The following analyses tested for differences between over-indebted and non-over-indebted consumers in the measures of interest (life satisfaction, emotional well-being, sleep, and health). As the two samples were not fully matched in terms of education, monthly income, employment status, and marital status (Table 1), we controlled for the effect of these variables by creating a propensity score indicating the predicted probability of being over-indebted vs. non-over-indebted, given these four potentially confounding variables.

#### Life Satisfaction

Using the valid data from 219 participants (104 over-indebted and 115 non-over-indebted), a repeated measures ANCOVA was conducted with Indebtedness Status (over-indebted, non-over-indebted) as a between-participants factor; Time of life satisfaction (current life satisfaction; predicted future life satisfaction) as a within-participants factor; and the propensity score as a covariate. The dependent variable was participant’s assessment of their own life satisfaction using ladder of Cantril (1965).

**Figure 1:**
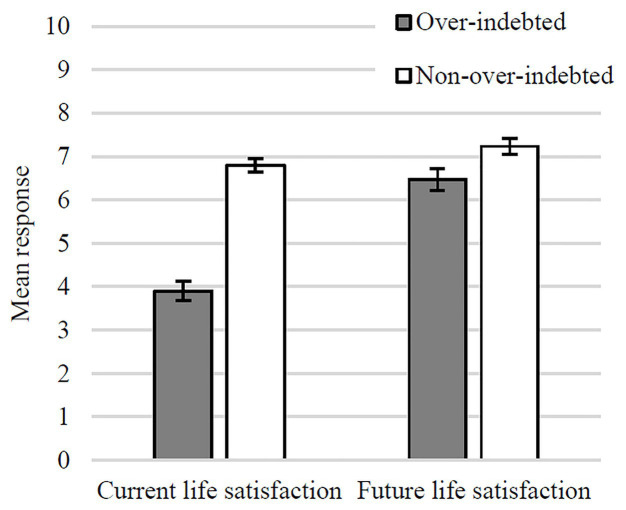
Mean evaluations of current and future life satisfaction (with SEs) in over-indebted and non-over-indebted groups.

The ANCOVA yielded two main effects and one interaction (Figure 1). A main effect of Indebtedness Status, *F*(1, 216) = 47.64, *p* < 0.001, *η_p_*^2^ = 0.18, such that over-indebted participants reported poorer overall life satisfaction [*M* = 5.18, *SE* = 0.19, 95% CI (4.81, 5.55)] than their non-over-indebted counterparts [*M* = 7.00, *SE* = 0.18, 95% CI (6.68, 7.37)]. A main effect of Time of life satisfaction, *F*(1, 216) = 4.46, *p* = 0.04, *η_p_*^2^ = 0.02, such that all participants reported higher predicted future life satisfaction [*M* = 6.85, *SE* = 0.16, 95% CI (6.55, 7.16)] than current life satisfaction [*M* = 5.35, *SE* = 0.13, 95% CI (5.10, 5.61)]. An interaction between Indebtedness Status and Time of life satisfaction, *F*(1, 216) = 35.35, *p* < 0.001, *η_p_*^2^ = 0.14, indicating that although non-over-indebted consumers reported an increase from current [*M* = 6.77, *SE* = 0.19, 95% CI (6.40, 7.14)] to predicted future life satisfaction [*M* = 7.28, *SE* = 0.22, 95% CI (6.81, 7.72)], *F*(1, 216) = 5.43, *p* = 0.021, *η_p_*^2^ = 0.02, for their over-indebted counterparts, this increase between current [*M* = 3.94, *SE* = 0.20, 95% CI (3.55, 4.33)] and predicted future life satisfaction [*M* = 6.43, *SE* = 0.24, 95% CI (5.96, 6.89)] was considerably steeper, *F*(1, 218) = 115.69, *p* < 001, *η_p_*^2^ = 0.35.

As expected, over-indebted consumers clearly presented overall lower levels of life satisfaction (i.e., across current and predicted future measures of life satisfaction) compared to non-over-indebted consumers. The current life satisfaction of over-indebted consumers is particularly low (below the midpoint of Cantril’s ladder). Interestingly, the reported interaction between current vs. predicted future life satisfaction and over-indebted vs. non-over-indebted consumers suggests that regardless of their difficult financial conditions, over-indebted consumers appear to believe in a better (financial) future as they anticipate a steeper increase in their predicted future life satisfaction.

#### Emotional Well-Being

Using the valid data from 209 participants (96 over-indebted and 113 non-over-indebted), a repeated measures ANCOVA was conducted with Time of the day (morning, afternoon, and evening) as a within-participants factor, Indebtedness Status (over-indebted, non-over-indebted) as a between-participants factor, and the propensity score as a covariate. The dependent variable was the net affect (composed of the subtraction of the mean negative emotions from the mean positive ones).

**Figure 2:**
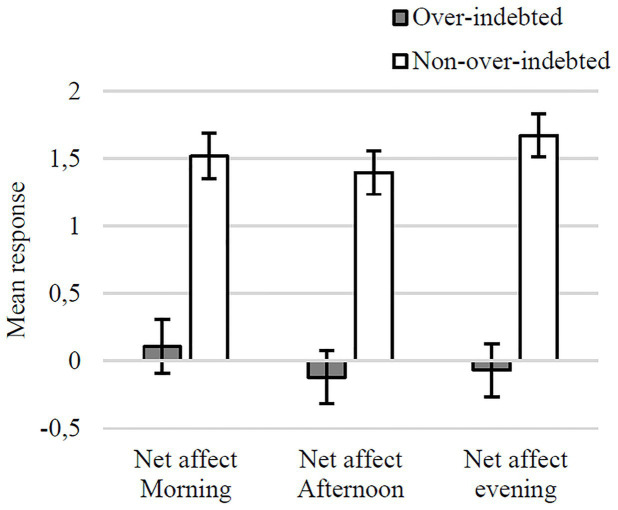
Mean evaluations of net affect on the previous day (with SEs) in over-indebted and non-over-indebted groups.

The ANCOVA yielded one main effect of Indebtedness Status, *F*(1, 206) = 29.91, *p* < 0.001, *η_p_*^2^ = 0.13, such that over-indebted consumers reported lower emotional well-being [*M* = −0.29, *SE* = 0.134 95% CI (−0.95, 0.37)] than their non-over-indebted counterparts [*M* = 1.15, *SE* = 0.43, 95% CI (0.30, 2.00)]. The interaction between indebtedness status and net affect suggests that the net affect of over-indebted consumers tended to deteriorate from morning to evening, while the net affect for non-over-indebted consumers tended to improve. However, this interaction did not reach statistical significance *F*(2, 412) = 2.39, *p* = 0.093, *η_p_*^2^ = 0.01 (Figure 2).

As expected, emotional well-being (operationalized in terms of net affect), was lower for over-indebted consumers compared to non-over-indebted consumers. Furthermore, over-indebted consumers experienced more negative emotions (relative to positive emotions) than non-over-indebted consumers, and this difference tends to become more pronounced from morning to evening.

#### Sleep

Using the valid data from 211 participants (100 over-indebted and 111 non-over-indebted), a repeated measures ANCOVA was conducted with Indebtedness Status (over-indebted, non-over-indebted) as a between-participants factor, Sleep (sleep time, sleep quality, and daytime dysfunction) as a within-participants factor, and with the propensity score as a covariate.

**Figure 3:**
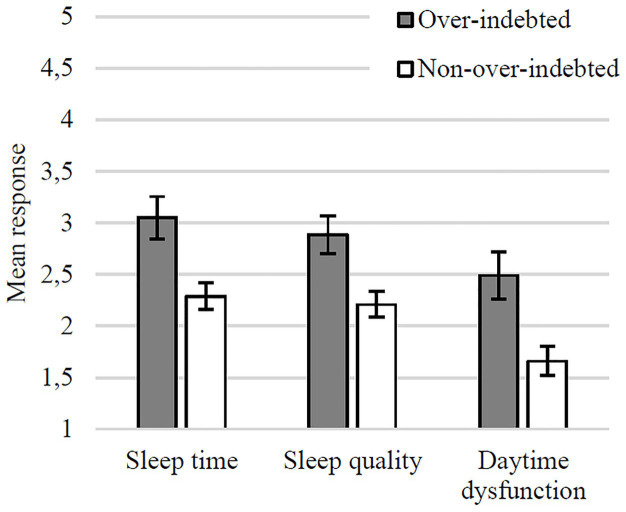
Mean evaluations of sleep time and quality and daytime dysfunction (with SEs) in over-indebted and non-over-indebted groups.

The ANCOVA yielded a main effect of Indebtedness Status, *F*(1, 208) = 54.60, *p* < 0.001, *η_p_*^2^ = 0.21, indicating that over-indebted consumers report worse sleep overall [*M* = 2.87, *SE* = 0.13, 95% CI (2.61, 3.11)] than non-over-indebted consumers [*M* = 2.13, *SE* = 0.16, 95% CI (1.81, 2.45)]. There was also a main effect of Sleep, *F*(2, 416) = 6.15, *p* = 0.002, *η_p_*^2^ = 0.03 [*M*_Sleep Time(ST)_ = 2.73, *SE*_ST_ = 0.14, 95% CI (2.45, 3.01), *M*_Sleep Quality(SQ)_ = 2.61, *SE*_SQ_ = 0.14, 95% CI (2.33, 2.89), and *M*_*Daytime Dysfunction*(DD)_ = 2.14, *SE*_DD_ = 0.14, 95% CI (1.86, 2.42)], suggesting lower levels of day time dysfunction compared to the sleep time and sleep quality components (Figure 3).

In short, over-indebtedness appears to have substantial negative effects on different aspects of sleep. Over-indebted consumers sleep less and worse than non-over-indebted consumers and have poorer daytime functioning.

#### Reported Health

Using the valid data from 226 participants (110 over-indebted and 116 non-over-indebted), a one-way ANCOVA was conducted with Indebtedness Status (over-indebted, non-over-indebted) as a between-participants variable and with the propensity score as covariate. The dependent variable was participants reported health.

The ANCOVA yielded a main effect of Indebtedness Status, *F*(1, 223) = 34.32, *p* < 0.001, *η_p_*^2^ = 0.13, such that over-indebted consumers reported poorer health [*M* = 3.00, *SE* = 0.08, 95% CI (2.85, 3.16)] than their non-over-indebted counterparts [*M* = 2.33, *SE* = 0.08, 95% CI (2.18, 2.48)].

Over-indebted consumers’ reported health was close to the rating scale point “fair” and significantly worse than non-over-indebted consumers’ health (which was closer to the point “good”).

#### Mediation Analysis

In this section, the results of the mediation analysis between indebtedness status (over-indebted and non-over-indebted) and the dependent variables, such as life satisfaction (current), global score of emotional well-being (aggregating for the morning, afternoon, and evening), and health and sleep (aggregating sleep time, sleep quality, and daytime dysfunction) are presented. We controlled for education, income, marital status, and employment status, *via* the propensity score created. Perceived control, financial anxiety, and financial well-being were included as possible mediators. The following analyses were performed using *MPlus* 7.2 (Muthén and Muthén, 1998–2012). Based on theoretical assumptions, significant correlations among mediators and among criterion variables were included in the model. To test the mediation hypotheses, bootstrap estimation was used with 5,000 subsamples to derive the 95% CI for the indirect effects (Preacher and Selig, 2012). The following fit indexes and criteria were used as indicative of a good model fit: the comparative fit index (CFI) and the Tucker-Lewis Index (TLI) higher than 0.95, the root mean square error of approximation (RMSEA) and standardized root mean residual (SRMR) lower than 0.08 (Hu and Bentler, 1999; Kline, 2011).

**Figure 4:**
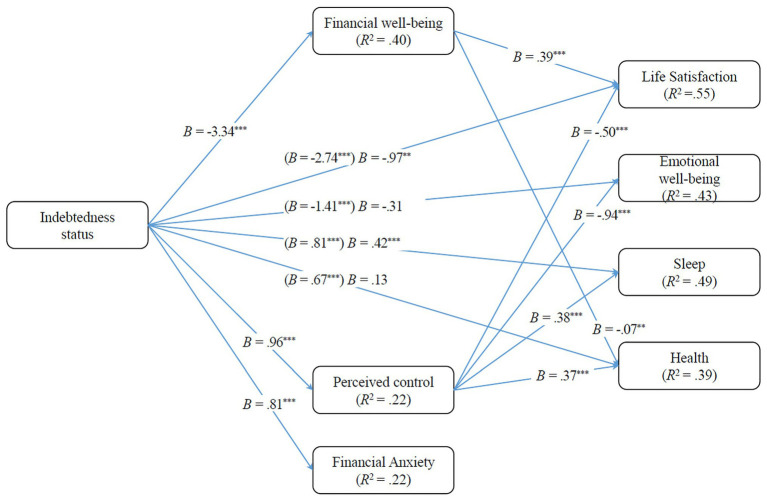
Mediation model for the effect of indebtedness status on life satisfaction, emotional well-being, sleep, and health *via* perceived control, financial well-being, and financial anxiety.

The multi-mediator path analysis model examining the indirect effects of indebtedness on life satisfaction, emotional well-being, health, and sleep, through perceived control, financial anxiety, and financial well-being, controlling for level of education, income, marital status, and employment status (through the propensity score) presented a good fit to the data: *χ*^2^(2) = 6.09, *p* = 0.048; CFI = 0.995; RMSEA = 0.095, 90% CI: (0.008, 0.185); SRMR = 0.013. The RMSEA index is slightly above the cutoff value of 0.08, but still under the 0.10 cutoff value for acceptable fit (Chen et al., 2008). Mode results are depicted in Figure 4.

Results revealed significant indirect effects of indebtedness on: (1) life satisfaction, through financial well-being, *B* = −1.30, *SE* = 0.30, *p* < 0.001, 95% CI: (−1.90, −0.76) and perceived control, *B* = −0.48, *SE* = 0.16, *p* = 0.002, 95% CI: (−1.61, −0.24); (2) emotional well-being, through perceived control, *B* = −0.91, *SE* = 0.17, *p* < 0.001, 95% CI: (−1.27, −0.60); (3) sleep quality, also through perceived control, *B* = 0.36, *SE* = 0.07, *p* < 0.001, 95% CI: (0.24, 0.49); and (4) reported health, through financial well-being, *B* = 0.22, *SE* = 0.08, *p* = 0.010, 95% CI: (0.05, 0.38) and perceived control, *B* = 0.36, *SE* = 0.08, *p* < 0.001, 95% CI: (0.21, 0.53).

More specifically, over-indebtedness was associated with: (1) lower levels of perceived control, which in turn predicted lower levels of life satisfaction, emotional well-being, sleep quality, and reported health; and (2) lower levels of financial well-being, which in turn predicted lower levels of life satisfaction and reported health. Although higher levels of financial anxiety were also predicted by over-indebtedness, financial anxiety did not emerge as a significant mediator in the model.

As shown in Figure 4, both total and direct effects of indebtedness on life satisfaction and sleep quality were significant, although the direct effects were lower than the total effect. In contrast, the direct effect of indebtedness on emotional well-being and on reported health was not significant. Thus, perceived control and financial well-being partially mediated the association between indebtedness status and life satisfaction, and fully mediated the association between indebtedness status and reported health. Finally, perceived control also fully mediated the association between indebtedness status and emotional well-being and partially mediated the association between indebtedness status and sleep quality. The propensity score did not predict any of the mediating or criterion variables.

Given the cross-sectional nature of the data, we tested four alternative models to exclude the possibility of other plausible pathways: (1) a model that was the “reverse” of our hypothesized model, examining the proposed criterion variables (i.e., life satisfaction, emotional well-being, sleep, and health) as predictors of indebtedness status, *via* the proposed mediators (i.e., perceived control, financial anxiety, and financial well-being; Alternative model 1); (2) a model examining indebtedness status as predictor, the proposed criterion variables as mediators, and the proposed mediators as criterion variables (Alternative model 2); and (3) a model examining the proposed mediators as predictors of the proposed criterion variables, *via* indebtedness status (Alternative model 3); and (4) a model examining perceived control as predictor of the proposed criterion variables, *via* indebtedness status, financial well-being, and financial anxiety (Alternative model 4). We then compared the fit of all models against one another to see which best fit the data. As shown in Table 2, the comparison of the fit indices of all models showed that the hypothesized model fit the data better than all four alternative models. In the next section, we thus focus on this model when discussing the mediational analysis results.

**Table 2:** Model fit comparison between the proposed model and the alternative models.

Model	χ^2^	*p* value	*df*	*χ*^2^/*df*	CFI	TLI	RMSEA	SRMR
Proposed model	6.09	0.048	2	3.04	0.995	0.916	0.095	0.013
Alternative model 1	11.28	<0.001	1	11.28	0.982	0.539	0.218	0.020
Alternative model 2	7.82	0.020	2	5.64	0.993	0.880	0.113	0.014
Alternative model 3	18.01	<0.001	3	10.63	0.979	0.792	0.151	0.027
Alternative model 4	56.24	<0.001	5	11.25	0.942	0.593	0.213	0.071

*χ*^2^/*df*, ratio of chi-square to degree of freedom; CFI, Comparative Fit Index; TLI, Tucker-Lewis Index; RMSEA, Root Mean Square Error of Approximation; and SRMR, Standardized Root Mean Square Residual.

## Discussion

This paper sought to assess and further explore the association of over-indebtedness to SWB, examining two of its components – life satisfaction and emotional well-being. This is an important issue for several reasons. First, although recent research has confirmed the negative outcomes of over-indebtedness in terms of mental and physical health (e.g., Emami, 2010; Gathergood, 2012; Angel, 2016), there is less research on the impact of debt on SWB. Second, although meta-analysis of Tay et al. (2017) found a negative association between debt and well-being, they did not distinguish between the two facets of well-being here considered. Furthermore, some prior research on well-being suggested that individuals rapidly adapt to life changing events and that most life circumstances have little influence on measures of SWB (Easterlin, 1995; Kahneman et al., 2004). Thus, the extent to which deteriorated life circumstances associated with over-indebtedness lead to decreased life satisfaction and/or emotional well-being is still an important research issue.

In our study, over-indebtedness was associated with lower life satisfaction, adding to the findings of Tay et al. (2017), but also with lower emotional well-being, a crucial component of SWB which has received less attention. Furthermore, the over-indebted consumers in our study were, for the most part, medium to long-term cases of over-indebtedness, who requested the assistance of a consumer defense NGO (DECO) following a lengthy period of financial hardship, and often as a last resort. This suggests that, in contrast with other life changing circumstances, the detrimental effects of over-indebtedness on life satisfaction and emotional well-being do not fade away, as proposed by the hedonic treadmill hypothesis (Brickman and Campbell, 1971). These results are in line with other findings that point to circumstantial changes that have more than just transitory effects. Among these changes are, for example, the effects of unemployment (often a main cause of indebtedness) and chronic pain (Lucas et al., 2004).

Our study further considered three potential mediators of the relationship between indebtedness status and the two facets of SWB: perceived control, financial anxiety, and financial well-being. The relationship between indebtedness status and life satisfaction was partly mediated by perceived control and financial well-being, that is, over-indebted consumers’ lower levels of perceived control and financial well-being partly explained their lower life satisfaction when compared to non-over-indebted consumers. As for emotional well-being, perceived control over one’s life fully explained the relationship between over-indebtedness and emotional well-being.

The partial mediation of financial well-being of the relationship between indebtedness and life satisfaction is in line with previous research (Diener et al., 1999; Kahneman, 1999; Tay et al., 2017) and provides some support for the first explanatory mechanism presented in the introduction, according to which life satisfaction is influenced by smaller life domains, one of them being financial well-being. Moreover, finding that financial well-being matters for life satisfaction but not for emotional well-being reaffirms the importance of considering separate measures of these two facets of well-being by confirming that they are different constructs with distinct determinants and consequences (Kahneman and Krueger, 2006).

The second explanation advanced in the introduction argues that a lack of financial resources limits the extent to which consumers may attain their goals, calling into question autonomy and self-control, which are crucial for well-being. The current results provide some support for this account by showing that over-indebtedness reduces consumers’ perceived self-control over their own lives. Reduced self-control then lowers both life satisfaction and emotional well-being.

This study also investigated whether indebtedness status predicted health and sleep quality. Confirming previous findings (e.g., Summers and Gutierrez, 2018), over-indebted consumers reported poorer overall health, worse sleep, and more sleep-related disturbances. These are important risk factors to consider. Self-ratings of health, for instance, have been found to be a strong predictor of mortality over the years (Mossey and Shapiro, 1982; Idler and Benyamini, 1997).

The mediating role of the same three variables (perceived control, financial anxiety, and financial well-being) on the impact of over-indebtedness on health and sleep were also explored. Perceived control and financial well-being emerged as the only significant mediators, with both fully mediating the relationship between indebtedness status and health, and perceived control partially mediating the relationship between indebtedness status and sleep quality.

It is noteworthy that perceived control emerged as a consistent mediator of the relationship between over-indebtedness and all dependent variables (life satisfaction, emotional well-being, health, and sleep). In other words, a lack of control over one’s life not only contributes to fully explaining the relationship between over-indebtedness and emotional well-being, but also partially explained the relationship between indebtedness status and life satisfaction. Furthermore, perceived control was also found to partially explain sleep quality and to fully explain reported overall health. These results are in line with recent findings by Białowolski et al. (2021), which show that financial control has a substantial impact on well-being (which is greater than being in a financially fragile situation). The authors found that financial control has a protective role on well-being through the promotion of (positive) emotional well-being outcomes and a protective one against emotional ill-being outcomes, which in turn translates into improved physical health and sleep.

Perceived control is a central motivator of individuals’ decision-making and behavior (e.g., Miller, 1979; Leotti et al., 2010; Higgins, 2012). It has been defined as “the belief that one can determine one’s own internal states and behavior, influence one’s environment, and/or bring about desired outcomes” (Wallston et al., 1987, p. 5). Individuals are highly motivated to believe they have control over their lives (e.g., Kelly, 1955; Burger and Cooper, 1979; Rothbaum et al., 1982) and to re-establish it in various ways. Thus, interventions aimed at promoting over-indebted consumers’ perceived control may be of relevance to improve their SWB.

Helzer and Jayawickreme (2015) explored the relationship between primary and secondary control strategies and the same two facets of well-being assessed in the present work. They defined primary control as the tendency to achieve mastery over circumstances *via* goal striving, and secondary control as the tendency to achieve mastery over circumstances *via* sense-making. Their findings indicated that primary control was more consistently associated with emotional well-being, whereas secondary control was associated with life satisfaction.

Taken together, the present findings and Helzer and Jayawickreme (2015) results suggest that the different strategies over-indebted consumers may use to re-establish control over their lives will differentially affect well-being. Thus, guidelines for interventions and the provision of support for over-indebted consumers should focus not only on primary control strategies, *via* measures that facilitate consumers’ efforts to recover from their severe financial difficulties (e.g., renegotiation of debt payment conditions), but also on secondary control strategies. In the latter case, this would involve helping over-indebted consumers to make sense of their challenging and complex social and financial situation (e.g., facing social discrimination, changing consumer habits) in order to better deal with it. This is particularly relevant for the cases in which increasing primary control might not be immediately feasible.

Finally, identifying over-indebtedness as a long-lasting threat to one’s SWB should be taken as a cautionary note for policymakers and practitioners alike. We suggest that the assessment of policies to fight over-indebtedness and empower consumers could include measures of life-satisfaction and emotional well-being. In other words, Government and NGO’s educational programs of financial guidance (e.g., financial literacy and financial decision-making courses) as well as specific legislation to protect and empower over-indebted consumers could include the improvement of consumers’ SWB among their standard goals.

### Limitations

Although our results unveil the stark consequences that over-indebtedness brings to one’s life, this study has several limitations that need to be considered.

First, a cross-sectional design was used, which prevents us from drawing strong conclusions that the individual differences in SWB (sleep quality and health) are due to over-indebtedness status. Nevertheless, the comparison of the proposed model with the alternative ones supported the hypothesized direction of effects over the other possible causal directions. Despite this, it is always possible that other variables (besides those we controlled for) also have predictive value over SWB.

Second, our measure of financial well-being was composed of a single item. Although single item measures have been used before (e.g., Johnson and Krueger, 2006; Switek, 2013), they fail to capture the multi-dimensional nature of financial well-being (Iannello et al., 2020). Future research should thus rely on multiple-item instruments that account for the different dimensions (cognitive, behavioral, materialistic, and relational) of financial well-being (e.g., Sorgente and Lanz, 2019; Iannello et al., 2020).

Third, our procedure classified all cases of over-indebted participants under the same conceptual umbrella, which is likely to be an oversimplification. In other words, not all over-indebted households should be considered equal (Ferreira et al., 2020). The diversity of risk factors of over-indebtedness strongly suggests that there are different over-indebted profiles associated to distinguishable causes (e.g., work loss, disease, low financial literacy, poor decision-making, and financial imprudence). A more fine-grained analysis of the impact of over-indebtedness on well-being should thus distinguish among these causes.

Fourth, although the present research acknowledged the multifaceted nature of well-being by measuring both life satisfaction and emotional well-being, a multidimensional perspective of well-being (Keyes et al., 2002; Iannello et al., 2020) further considers the concept of psychological well-being, which entails the perception of engagement with existential life challenges (Keyes et al., 2002) and is often assessed through the concept of flourishing (Diener et al., 2010). Hence, in order to better evaluate the impact of over-indebtedness on the multiple dimensions of well-being, future research should also include operationalizations of psychological well-being (Ryff and Keyes, 1995).

Future research should ideally replicate these findings using better and more comprehensive measures of well-being and using new matched samples of over-indebted and non-over-indebted households. Furthermore, longitudinal designs with at least two waves of data collection are crucial to clarify causal links and more clearly disentangle alternative mediating directions.

## Conclusion

In sum, the reported findings (and their limitations) should not be evaluated in isolation but rather as another research effort, contributing to a literature concerning the effects of debt on consumers’ well-being. Our results are well-aligned with prior research on the psychological and physical implications of over-indebtedness (replicating several previous findings) and provide some new insights in terms of the underlying mechanisms that link indebtedness to well-being. Moreover, to our knowledge, these are among the first findings specifically focusing on over-indebtedness and different facets of well-being (but see also Angel, 2016; Białowolski et al., 2019) and the first within the Portuguese context. Hopefully, they will contribute to set the stage for further research on these mechanisms and their boundary conditions.

## Data Availability Statement

The raw data supporting the conclusions of this article will be made available by the authors, without undue reservation.

## Ethics Statement

The studies involving human participants were reviewed and approved by Ethics committee of the Faculty of Psychology, University of Lisbon. The participants provided their written informed consent to participate in this study.

## Author Contributions

MF, FA, and JS contributed equally to the conceptualization, data collection and analysis, manuscript writing, and thus sharing first co-authorship. DP, MH, and CS provided the indispensable analysis and inputs on results and discussion. All authors contributed to the article and approved the submitted version.

### Conflict of Interest

The authors declare that the research was conducted in the absence of any commercial or financial relationships that could be construed as a potential conflict of interest.
